# Very Suggestive and Promising

**Published:** 1887-12

**Authors:** 


### *VERY SUGGESTIVE AND PROMISING.*

The alumni of the Jefferson Medical College have at last taken a decided step to show their dissatisfaction with the want of desire on the part of the management to adopt a higher grade of instruction in that school. A few days ago, the executive committee of the Alumni Association sent the following recommendations to the faculty:

WHEREAS, It having come to the knowledge of this executive committee of the Alumni Association, that four students have gone to the University of Pennsylvania to pursue their medical education at the recommendation of the Alumni of this College, on the ground that Jefferson Medical College did not provide a three-years' graded course, and did not furnish clinics or instruction on the special branches of medicine and surgery—

*Be it Resolved,* That the executive committee, having the best interest of our Alma Mater at heart, respectfully announce these facts to the faculty, that they may take such action as they deem best to overcome this apparent growing dissatisfaction of our alumni.

Surely, the suggestiveness of such a step, on the part of a college's own alumni, must be apparent, and speaks well for the future of our medical schools and their graduates. The call for more work from the student is becoming louder every year, and the end cannot now be far away.

